# Lesion Morphology and Technical Success in Diabetic vs. Non-Diabetic Patients with Chronic Limb-Threatening Ischemia Undergoing Endovascular Treatment: A Retrospective Single-Center Study

**DOI:** 10.3390/medicina62071301

**Published:** 2026-07-06

**Authors:** Anca Dumitrescu-Bordianu, Genoveva Livia Baroi, Cristina Dascălu, Radu Florin Popa, Lucian Iacob, Alin Horațiu Nedelcu, Paula Cristina Morariu, Maria Mihaela Godun, Anton Knieling, Ioana-Cezara Caba, Bogdan Mihai Diaconescu, Bogdan Huzum, Bogdan Caba, Mariana Floria

**Affiliations:** 1 Faculty of Medicine, Grigore T. Popa University of Medicine and Pharmacy, 700115 Iași, Romania; bordianu.anca@d.umfiasi.ro (A.D.-B.); livia.baroi@umfiasi.ro (G.L.B.); cristina.dascalu@umfiasi.ro (C.D.); radu.popa@umfiasi.ro (R.F.P.); luciiacob@gmail.com (L.I.); alin.nedelcu@umfiasi.ro (A.H.N.); morariu.paula-cristina@email.umfiasi.ro (P.C.M.); godun.maria-mihaela@d.umfiasi.ro (M.M.G.); anton.knieling@umfiasi.ro (A.K.); mihail.diaconescu@umfiasi.ro (B.M.D.); bogdan.huzum@umfiasi.ro (B.H.); floria.mariana@umfiasi.ro (M.F.); 2 Faculty of Pharmacy, Grigore T. Popa University of Medicine and Pharmacy, 700115 Iași, Romania; ioana-cezara.caba@umfiasi.ro; 3 Faculty of Medical Bioengineering, Grigore T. Popa University of Medicine and Pharmacy, 700020 Iași, Romania

**Keywords:** chronic limb-threatening ischemia, diabetes mellitus, endovascular revascularization, lesion morphology, infrapopliteal disease, technical success

## Abstract

*Background and Objectives*: Chronic limb-threatening ischemia (CLTI) represents the most advanced stage of peripheral arterial disease and is associated with an increased risk of major amputation, particularly in patients with diabetes, in whom arterial disease frequently presents with distinct anatomical patterns. While global scores are widely used, the impact of lesion morphology on procedural outcomes is less clearly defined. This study aims to evaluate the distribution of lesion types (stenoses versus occlusions) and their association with the technical success of endovascular revascularization in CLTI, with a focus on differences between diabetic and non-diabetic patients. *Materials and Methods*: This retrospective study included 136 consecutive patients with chronic limb-threatening ischemia (CLTI) who underwent endovascular revascularization at the Vascular Surgery Department of the “Sf. Spiridon” Clinical Emergency Hospital, Iași, Romania, between May 2021 and May 2023, comprising 85 (62.5%) diabetic and 51 (37.5%) non-diabetic patients. The mean age of the study population was 69.9 ± 9.7 years, and 38 (27.9%) patients were female. Baseline demographic characteristics and major comorbidities were broadly comparable between the two groups. Angiographic lesions were classified according to the degree of luminal narrowing as non-significant stenosis (<50%), significant stenosis (≥50%), or occlusion (complete absence of contrast flow) at the femoropopliteal and infrapopliteal levels. Technical success was defined as restoration of “inline” flow to the leg with residual stenosis < 30%. Patients treated with stenting and those undergoing infrapopliteal angioplasty received short-term (up to 6 weeks) dual antiplatelet therapy. Associations between lesion morphology and procedural outcomes were assessed using appropriate statistical methods. *Results*: Diabetic patients had more frequent stenotic lesions (55.3% vs. 35.3%, *p* = 0.024) at the infrapopliteal level, whereas non-diabetic patients had more frequent occlusive lesions (96.1% vs. 83.5%, *p* = 0.028) at the femoropopliteal level. These differences were associated with procedural outcomes. Occlusive lesions, especially at key anatomical segments, were associated with an increased risk of technical failure, whereas stenotic lesions were more frequently associated with successful endovascular revascularization. *Conclusions*: Morphological characteristics of lesions play a critical role in determining the success of procedures in CLTI. Stenotic lesions in diabetic patients suggest a distinct vascular phenotype that may influence revascularization strategies. The results highlight the importance of detailed lesion assessment in planning interventions, beyond the use of conventional scores.

## 1. Introduction

Chronic limb-threatening ischemia (CLTI) is the most severe form of peripheral artery disease, associated with high rates of limb loss and mortality, with reported 1-year amputation rates of 15–25% and mortality approaching 20–25%, particularly in patients with diabetes mellitus [1,2,3]. Data from BEST-CLI show that diabetes mellitus is independently associated with an increased risk of amputation and mortality after revascularization in patients with CLTI (including both endovascular and surgical approaches), likely driven by greater injury severity and increased prevalence of comorbidities, including heart failure, coronary artery disease, and chronic kidney disease [4].

Management of therapeutic strategies in modern medicine emphasizes revascularization using endovascular techniques, guided by anatomical and clinical assessment tools such as the WIFI, GLASS, or Bollinger scoring systems [1,3,5]. However, although these systems provide an assessment of disease severity and the complexity of revascularization, they focus primarily on total or segmental burden rather than on intrinsic morphological features of arterial lesions. In difficult clinical situations requiring repeated or advanced infrapopliteal interventions, alternative strategies, such as venous bypass grafting or deep venous arterialization, may be considered [6].

Diabetes alters the distribution and nature of peripheral arterial disease through endothelial dysfunction, chronic inflammation, oxidative stress, and medial arterial calcification, resulting in diffuse atherosclerosis; impaired collateral formation; and a predominance of distal, infrapopliteal involvement, often associated with concomitant microvascular dysfunction [1,3]. In this context, lesion morphology—particularly the distinction between stenotic and occlusive disease—may have important implications for procedural feasibility and outcomes after endovascular revascularization [7,8]. Occlusive lesions are associated with increased technical complexity and lower procedural success rates, whereas stenotic lesions may be more amenable to endovascular treatment, especially in small-caliber distal vessels [8]. Despite this, lesion type as an independent determinant of outcome remains insufficiently explored. Additionally, comparative analyses between diabetic and non-diabetic patients with CLTI are necessary. In patients with chronic limb-threatening ischemia, the combination of optimal drug treatment with endovascular interventions leads to superior results for limb salvage and reduction in complications. Therefore, the present study aims to evaluate the distribution of lesion morphology and its impact on technical success in endovascular treatment, with a specific focus on differences related to diabetic status.

## 2. Materials and Methods

### 2.1. Clinical Analysis

We conducted a retrospective, single-center study in a tertiary care center. Inclusion criteria comprised consecutive adult patients (≥18 years) with chronic limb-threatening ischemia (CLTI) who underwent endovascular revascularization between May 2021 and May 2023 at the Vascular Surgery Department of the “Sf. Spiridon” Emergency Clinical Hospital, Iași, Romania. Baseline demographic characteristics, cardiovascular risk factors, and comorbidities were recorded. Angiographic evaluation was performed using digital subtraction angiography. Exclusion criteria included acute limb ischemia, Raynaud’s phenomenon, aortic dissection, previous lower-limb bypass surgery, and other non-atherosclerotic causes of lower-limb ischemia. Among the 201 patients who underwent lower-limb angiography/endovascular procedures during the study period, 57 were excluded because of diagnostic angiography without a treatable lesion (*n* = 42) or isolated aorto-iliac disease (*n* = 15). Among the remaining 144 eligible infrainguinal CLTI patients, 8 were excluded due to incomplete clinical or angiographic data, resulting in a final study cohort of 136 patients (85 diabetic and 51 non-diabetic).

**Figure 1:**
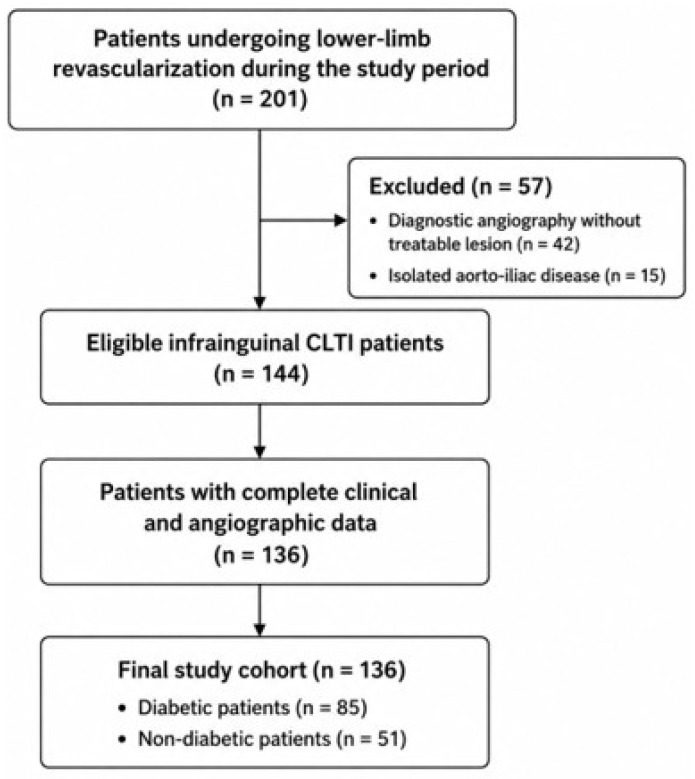
Flowchart illustrating patient selection and study cohort formation.

The patient selection process is summarized in Figure 1.

This study was approved by the institutional Ethics Committee of St. Spiridon County Emergency Hospital (no. 90/3 October 2023) and conducted in accordance with the Declaration of Helsinki.

Standard techniques such as balloon angioplasty with stenting were used for endovascular procedures. In complex or occlusive lesions, guidewires and support catheters were used to advance across the lesions. Lesions were classified according to the degree of luminal narrowing (<50%, ≥50%, or occlusion), in line with established angiographic assessment frameworks such as the Bollinger scoring system, and analyzed at the femoropopliteal and infrapopliteal levels. Technical success was defined as restoration of inline flow to the foot with <30% residual stenosis on completion angiography. Technical failure was defined as the inability to achieve inline flow to the foot and/or the presence of residual stenosis ≥ 30% despite endovascular treatment. We adopted the “easiest-first” strategy, which means prioritizing revascularization of the technically most feasible artery, rather than following the angiosome concept. The aim was to restore direct flow to the foot, followed by reassessment of distal perfusion, and to decide the need for additional interventions [9].

All stented patients and those with infrapopliteal PTA (percutaneous transluminal angioplasty) received dual antiplatelet therapy (DAPT) for up to 6 weeks, with a loading dose on the day of the procedure for those not on antiplatelets preoperatively. All patients received lifelong single antiplatelet therapy after an initial 6 weeks of DAPT. Patients with stents who had atrial fibrillation and were previously treated with oral anticoagulants received NOACs in combination with a single antiplatelet agent; in elderly patients aged ≥75 years, simplified therapeutic regimens were preferred, with an individualized approach based on bleeding risk.

### 2.2. Statistical Analysis

All analyses were performed by SPSS (version 29, IBM Corp., Armonk, NY, USA). Data were expressed as mean ± standard deviation, and categorical variables as absolute numbers and percentages. Variables associated with technical success were evaluated using univariate logistic regression, and odds ratios (ORs) with 95% confidence intervals (CIs) were reported. Group comparisons were performed using Student’s *t*-test, the Mann–Whitney U test for continuous variables, and the chi-square or Fisher’s exact test for categorical variables, as appropriate. Correlations between scoring systems were assessed using Spearman’s rank correlation coefficient. Agreement between classifications was evaluated using weighted kappa statistics. Logistic regression analysis was performed to explore predictors of technical failure. Model performance was assessed using the Hosmer–Lemeshow goodness-of-fit test and Nagelkerke R^2^. Statistical significance was defined as *p* < 0.05.

Sample size was estimated using OpenEpi for a finite population. Considering the 201 patients screened during the study period, a 95% confidence level, a 5% margin of error, and an expected proportion of 50% to provide the most conservative estimate, the minimum required sample size was 133 patients. The final cohort included 136 patients, thus meeting the estimated sample size requirement. The formula used was *n* = [N × Z^2^ × p × (1 − p)]/[d^2^ × (N − 1) + Z^2^ × p × (1 − p)], where N is the finite population size, Z is the standard normal deviate, p is the expected proportion, and d is the margin of error.

## 3. Results

A total of 136 patients with chronic limb-threatening ischemia (CLTI) undergoing endovascular revascularization were included, of whom 85 (62.5%) were diabetic and 51 (37.5%) were non-diabetic. Baseline demographic characteristics, cardiovascular risk factors, and major comorbidities are summarized in Table 1. Overall, baseline characteristics were comparable between groups. Although hypertension was more frequent among diabetic patients (85.9% vs. 74.5%), the difference did not reach statistical significance (*p* = 0.097).

The morphology of lesions differed significantly between groups. Patients with diabetes more frequently presented with ≥50% stenosis (55.3% vs. 35.3%, *p* = 0.024), whereas non-diabetic patients more often had occlusive disease (96.1% vs. 83.5%, *p* = 0.028). The proportion of <50% stenosis was similar between groups (45.1% vs. 45.9%, *p* = NS). At the global level, the distribution of lesion types according to diabetic status is summarized in Table 2. At the segmental level, the most pronounced difference was observed in the superficial femoral artery. Diabetic patients more frequently exhibited > 50% stenosis (34.1% vs. 15.7%), whereas non-diabetic patients had a higher prevalence of occlusions (47.1% vs. 28.2%; overall distribution, *p* < 0.001). Segmental grouping was performed by aggregating femoropopliteal and infrapopliteal (ATA/PTA/TPT) arterial segments (Table 3).

Segmental study indicated that, in diabetes patients, infrapopliteal illness was typified by diffuse, multilevel stenotic involvement with generally maintained artery continuity, whereas non-diabetic patients more frequently exhibited localized or long-segment occlusions. In the tibial area, stenotic lesions were more evident in diabetic patients, and occlusive lesions were more frequently encountered in non-diabetic patients, which may suggest a distinct phenotype of distal disease. Consistent with these findings, diabetic patients also demonstrated lower whole-limb (30.07 ± 16.34 vs. 36.78 ± 16.09, *p* = 0.018) and infrapopliteal Bollinger scores (20.53 ± 15.88 vs. 26.86 ± 13.93, *p* = 0.015) than non-diabetic patients, further supporting the observed differences in lesion morphology.

**Figure 2:**
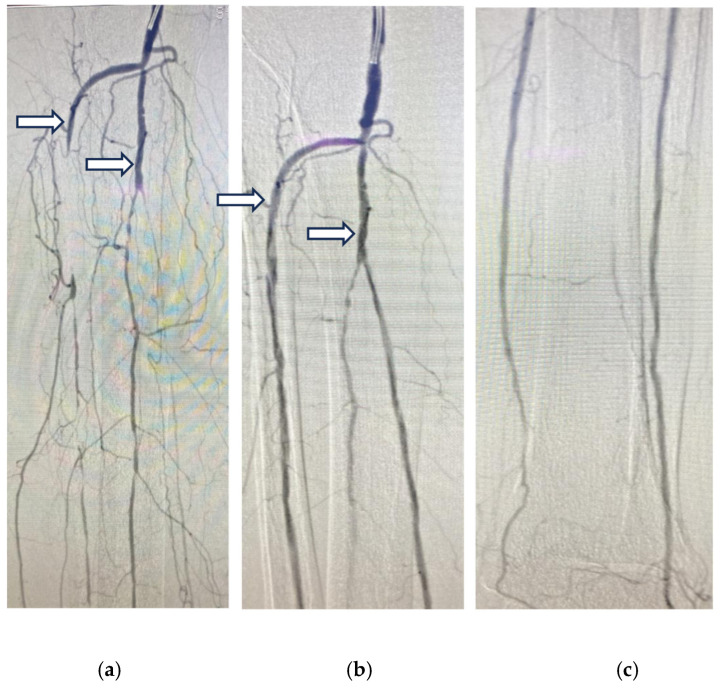
Diagnostic angiography demonstrating infrapopliteal disease and treatment in a diabetic patient. White arrows in panel (**a**) indicate the main occlusive and stenotic lesions. (**a**) Preprocedural angiography—serial occlusions and >50% stenoses of the anterior and posterior tibial arteries with poor distal run-off. (**b**) Lesion crossing and balloon angioplasty of the anterior tibial and posterior tibial arteries (3.0 and 2.5 mm balloons). (**c**) Final angiography—inline flow to the foot and improved distal run-off.

**Figure 3:**
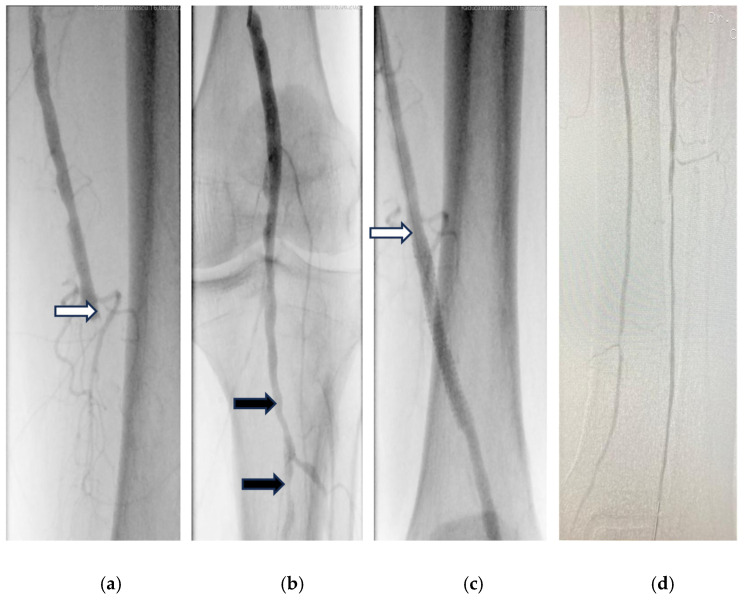
Diagnostic angiography illustrating stepwise endovascular treatment of SFA in a non-diabetic patient. White arrows indicate the chronic total occlusion (CTO) of the SFA and the implanted stent after recanalization, whereas black arrows indicating the popliteal and tibioperoneal trunk lesions treated during the procedure. (**a**) CTO of the SFA within the adductor canal. (**b**) Lesion crossing and initial recanalization of the occluded segment with treatment of PA and TPT lesions. (**c**) Balloon angioplasty of SFA (⌀ 5.0 and 6.0 mm) with restoration of vessel patency. (**d**) Final angiographic results after SFA stenting (⌀ 7 mm × 60 mm), demonstrating restoration of inline flow to the foot through patent ATA and PTA.

In Figure 2 and Figure 3, the angiographic results with lesion morphology and the results of endovascular revascularization in both diabetic and non-diabetic patients are represented.

These segmental differences in lesion morphology provided the basis for further analysis of their impact on procedural outcomes. The primary outcome was technical success, which was achieved in 101 of 136 procedures (74.3%), whereas technical failure occurred in 35 cases (25.7%). Technical failure was primarily related to the inability to cross the target lesion (wire- or balloon-uncrossable), most commonly because of severe calcification, chronic total occlusions, or complex multilevel arterial disease. Only hemodynamically significant lesions (≥50% stenosis and occlusions) were included in the regression model. Several lesion-related variables showed significant associations with technical success. Stent use was more frequent in technically successful procedures than in failures (27.7% vs. 8.6%, *p* = 0.020), corresponding to a lower risk of technical failure (OR 0.244, 95% CI 0.069–0.863). The strongest associations are summarized in Table 4.

A more detailed segmental analysis showed that occlusive disease at the superficial femoral artery, popliteal artery, and tibioperoneal trunk significantly increased the likelihood of technical failure, with the highest effect observed for popliteal occlusion (OR 4.650, *p* = 0.026). At the superficial femoral artery level, failure cases more frequently showed occlusion (54.3% vs. 28.7%) and >50% stenosis (28.6% vs. 26.7%), corresponding to ORs of 2.948 and 3.884, respectively. At the popliteal level, occlusions were more frequent among failed treatments (28.6% vs. 7.9%). A similar pattern was observed at the tibioperoneal trunk (28.6% vs. 8.9%; OR 4.089, *p* = 0.020), supporting the association between distal occlusive disease and technical failure.

In diabetic patients, superficial femoral artery morphology remained significantly associated with failure (*p* = 0.024), whereas in non-diabetic patients, popliteal occlusion emerged as the strongest predictor of unsuccessful revascularization (42.9% vs. 8.1%; OR 8.500, *p* = 0.029).

In 74.3% of cases, overall technical success was achieved, with no significant difference observed between diabetic and non-diabetic patients. Procedural outcomes according to diabetic status are summarized in Table 5.

Occlusive lesions, especially in important anatomical segments, were associated with a higher risk of technical failure. Correlation analysis revealed a moderate connection between Bollinger and GLASS scores at the whole-limb level (ρ ≈ 0.64, *p* < 0.001), with more robust correlations identified at the infrapopliteal level (ρ = 0.71, *p* < 0.001). Correlations between WIfI and angiographic scores were weaker but remained statistically significant.

## 4. Discussion

CLTI is a complex condition requiring an integrated assessment of limb severity, anatomical disease burden, and patient risk, as outlined in the Global Vascular Guide [1]. Current guidelines support revascularization as the cornerstone of management, with an increasing role for endovascular approaches [2]. Endovascular revascularization should be the preferred treatment to restore perfusion to the lower extremity in patients with chronic limb-threatening ischemia (CLTI), and studies that evaluate the permeability of reinterventions even in infrapopliteal segments show they also have acceptable 30-day mortality and complication rates [6]. Anatomical characteristics remain central to both strategy selection and procedural success, as highlighted in the Global Vascular Guidelines, where lesion characteristics are integrated through the GLASS classification, and supported by contemporary evidence from trials such as BEST-CLI and BASIL [5,10].

Our study demonstrates that lesion morphology differs substantially between diabetic and non-diabetic patients with CLTI undergoing endovascular revascularization, with clear implications for procedural complexity. In our cohort, diabetic patients more often presented with diffuse infrapopliteal stenotic disease, whereas non-diabetic patients more frequently presented with occlusive lesions, particularly at the femoropopliteal level. This distribution may help explain the technical challenges encountered in the first group and underline the importance of careful preoperative planning.

To provide additional context regarding disease severity, we also evaluated the angiographic burden of atherosclerosis using the Bollinger score. Diabetic patients showed lower whole-limb and infrapopliteal Bollinger scores than non-diabetic patients, supporting the concept that diabetic arteriopathy is characterized by a predominance of diffuse stenotic lesions rather than extensive occlusive disease.

Beyond confirming previously reported differences in disease distribution, our findings provide additional insight into angiographic lesion morphology and its potential impact on endovascular strategy. This detailed morphological characterization may support a more individualized approach to procedural planning in patients with CLTI.

The present findings are consistent with previous observations that diabetic arterial disease is predominantly distal and diffuse, with a higher burden of infrapopliteal involvement and fewer focal occlusions [1,3]. This pattern has been linked to medial arterial calcification and microvascular remodeling, contributing to the development of a characteristic distal disease phenotype in diabetes [7,11]. Moreover, the concept of distinct arterial distribution patterns in CLTI, including small-vessel and distal involvement, has been increasingly recognized in the contemporary literature [11].

From a morphological perspective, infrapopliteal lesions in CLTI are characterized by extensive longitudinal involvement, reduced vessel diameter, and high complexity [8]. These characteristics were reflected in our diabetic cohort, where diffuse stenotic disease predominated at the tibial level. Importantly, prior studies have demonstrated that the burden of infrapopliteal disease plays a major role in clinical outcomes, regardless of diabetic status, reinforcing the prognostic relevance of distal disease distribution [11,12].

Lesion morphology is considered a determinant of procedural outcome in our analysis. Occlusive disease in critical anatomical segments (popliteal artery and tibioperoneal trunk) is associated with a high rate of technical failure. This finding is consistent with previous evidence that lesion complexity, including chronic total occlusions and distal vessel involvement, significantly reduces the likelihood of successful endovascular revascularization [5,13]. In addition, arterial calcification represents a major factor influencing procedural difficulty and technical success in peripheral interventions [7].

In the superficial femoral artery, both significant stenosis and occlusion were associated with an increased risk of technical failure, demonstrating the impact of femoropopliteal disease as a determinant of procedural complexity. The strong association observed with popliteal artery occlusion underscores the hemodynamic relevance of this segment, where flow limitation may affect distal perfusion and procedural success [8,13].

The use of stents has led to improved technical success, suggesting that a lesion scaffold may partially compensate for the negative impact of lesion morphology. This observation is in line with contemporary endovascular strategies aimed at optimizing luminal gain and procedural stability in difficult lesions [5].

The comparative analysis of early outcomes after endovascular revascularization in patients with chronic limb-threatening ischemia showed no statistically significant differences between diabetic and non-diabetic patients. Technical success was achieved in 37 non-diabetic patients (72.5%) and in 64 diabetic patients (75.3%), with a comparable success rate between groups (*p* = 0.71). Similarly, the need for conversion to bypass was identical in both cohorts, occurring in 6 non-diabetic patients (11.8%) and 10 diabetic patients (11.8%) (*p* > 0.05).

Although major amputation was numerically more frequent among diabetic patients, affecting 8 cases (9.4%) compared with 2 cases (3.9%) in the non-diabetic group, this difference did not reach statistical significance (*p* = 0.32). In-hospital mortality was low in both groups, with one death among non-diabetic patients (2.0%) and three deaths among diabetic patients (3.5%), again without a statistically significant difference (*p* > 0.05).

Overall, these findings suggest that diabetes mellitus was not associated with significantly different immediate procedural or in-hospital outcomes following endovascular revascularization in this cohort of patients with chronic limb-threatening ischemia. However, the higher numerical rate of major amputation in diabetic patients may indicate a clinically relevant trend that should be interpreted cautiously, particularly in the context of sample size and disease severity.

Importantly, despite clear differences in lesion morphology between diabetic and non-diabetic patients, overall technical success did not differ significantly between groups. These results may suggest that diabetes may influence the anatomical pattern of the disease, and the outcome of the procedure determines the specific characteristics of the lesion at the segmental level, rather than the diabetic status [13]. Also, at the infrapopliteal level, the moderate correlation between the Bollinger and GLASS scores supports the internal consistency of angiographic burden assessment across different classification systems [14,15,16]. The effect of diabetes mellitus on clinical outcomes such as major amputation, mortality, or major adverse events in the limb does not seem to be determined exclusively by the severity of ischemia but also by the particularities of the clinical presentation and the association of other comorbidities [4]. The weaker correlation between WIfI and angiographic scores suggests that clinical severity and anatomical disease burden represent complementary, rather than overlapping, dimensions of CLTI, as previously reported [3]. Accordingly, the management of CLTI with infrapopliteal involvement requires an individualized, evidence-based revascularization strategy that integrates hemodynamic and imaging assessment with the use of standardized frameworks such as WIfI, PLAN, and GLASS for staging and procedural planning [9].

Data also highlight the complexity of antithrombotic management following surgical revascularization, where graft patency often requires long-term antiplatelet or anticoagulant therapy. Endovascular revascularization, on the other hand, does not use grafts and is typically managed with antiplatelet-based regimens, with treatment intensity tailored according to individual thrombotic and bleeding risk [17].

The approach to post-interventional antithrombotic therapy varies significantly between studies, with an increased trend towards the use of dual antiplatelet therapy (DAPT) after endovascular interventions, for a variable period of time, with the aim of reducing the risk of stent thrombosis and restenosis, depending on the type of intervention and the bleeding risk [18,19].

In carefully selected patients with well-demarcated dry gangrene who are not candidates for revascularization or major surgery, a conservative strategy allowing spontaneous autoamputation may be considered under close clinical surveillance. However, this approach should be reserved for highly selected cases and should not delay surgical intervention when infection or wet gangrene is present [20].

Although our study focused on atherosclerotic CLTI, distal tissue necrosis may also occur in non-atherosclerotic conditions. Vasopressor-induced peripheral ischemia represents a rare but severe complication of prolonged vasopressor therapy in critically ill patients and requires early recognition and multidisciplinary management to minimize tissue loss [21]. Overall, the management of patients with chronic limb-threatening ischemia (CLTI) is complex and multidimensional, requiring an integrated, multidisciplinary approach that includes medical therapy, wound care, vascular assessment, and revascularization procedures, to optimize clinical outcomes and salvage the limb [22].

## 5. Limitations

This study has several limitations. First, its retrospective, single-center design may limit the generalizability of the findings and introduce selection bias. Second, the relatively small sample size may have limited the statistical power for some subgroup analyses. Therefore, larger prospective multicenter studies are needed to confirm our findings.

This study was conducted in a vascular surgery department, with the primary focus on angiographic lesion morphology and the technical success of endovascular treatment rather than diabetes management. Consequently, detailed information regarding glycemic control, including HbA1c values, duration of diabetes, and antidiabetic therapy, was not consistently available because of the retrospective study design and could not be reliably analyzed.

## 6. Conclusions

In this cohort of patients with chronic limb-threatening ischemia, diabetic and non-diabetic individuals exhibited distinct patterns of arterial disease, with diabetes associated with more diffuse infrapopliteal involvement. Although overall technical success was similar across groups, specific lesion characteristics were strongly associated with procedural failure. Our study confirmed the need for lesion-oriented planning with particular attention to occlusions at key anatomical segments and a targeted infrapopliteal revascularization strategy to improve distal run-off. The management of CLTI must be approached in an integrated manner, with drug therapy indispensable both before and after endovascular interventions, and maximum efficiency achieved by combining revascularization with optimization of systemic pharmacotherapy.

## Figures and Tables

**Table 1:** Baseline demographic characteristics, cardiovascular risk factors, and comorbidities of the study population.

Parameter	NDM Group (*n* = 51)	DM Group (*n* = 85)	*p*-Value
Female, n (%)	12 (23.5)	27 (31.8)	0.304
Urban residence, n (%)	25 (49.0)	62 (72.9)	0.005 **
Age, years (mean ± sd)	69.78 ± 11.38	69.89 ± 8.57	0.737
Hypertension, n (%)	38 (74.5)	73 (85.9)	0.097
Obesity, n (%)	4 (7.8)	13 (15.3)	0.203
Heart failure, n (%)	17 (33.3)	24 (28.2)	0.531
Atrial fibrillation, n (%)	15 (29.4)	16 (18.8)	0.154
Prior mi, n (%)	2 (3.9)	7 (8.2)	0.483
Chronic coronary syndrome, n (%)	8 (15.7)	17 (20.0)	0.530
Cardiomyopathy, n (%)	1 (2.0)	5 (5.9)	0.410
Dilated cardiomyopathy, n (%)	4 (7.8)	4 (4.7)	0.473
Chronic kidney disease, n (%)	7 (13.7)	16 (18.8)	0.443
Prior stroke, n (%)	2 (3.9)	10 (11.9)	0.210

Abbreviations: NDM: non-diabetic patients; DM: diabetic patients; **—statistically highly significant.

**Table 2:** Distribution of lesion types according to diabetic status.

Lesion Type	Non-Diabetic (*n* = 51)	Diabetic (*n* = 85)	*p*-Value
<50% stenosis	23 (45.1%)	39 (45.9%)	NS
≥50% stenosis	18 (35.3%)	47 (55.3%)	0.024
Occlusion	49 (96.1%)	71 (83.5%)	0.028

**Note:** Lesion categories were not mutually exclusive; multiple lesion types could be present in the same patient.

**Table 3:** Segmental distribution of lesion morphology in diabetic versus non-diabetic patients.

Variable	Non-Diabetic (*n* = 51)	Diabetic (*n* = 85)	*p*-Value
Femoropopliteal < 50% stenosis	20 (39.2%)	27 (31.8%)	NS
Femoropopliteal ≥ 50% stenosis	12 (23.5%)	43 (50.6%)	<0.01
Femoropopliteal occlusion	26 (51.0%)	32 (37.6%)	0.03
Infrapopliteal < 50% stenosis	6 (11.8%)	8 (9.4%)	NS
Infrapopliteal ≥ 50% stenosis	25 (49.0%)	53 (62.4%)	0.02
Infrapopliteal occlusion	32 (62.7%)	47 (55.3%)	NS

**Note:** Lesion categories were not mutually exclusive and may coexist within the same arterial segment. NS—Not Significant.

**Table 4:** Association between lesion morphology and technical success in endovascular revascularization.

Variable	OR (95% CI)	*p*-Value	Interpretation
Stent present	0.244 (0.069–0.863)	0.020	Protective
SFA > 50% stenosis	3.884 (1.483–10.0)	0.029	Risk factor
SFA occlusion	2.948 (1.335–6.513)	0.029	Risk factor
PA occlusion	4.650 (1.661–13.015)	0.026	Risk factor
TPT occlusion	4.089 (1.499–11.150)	0.020	Risk factor

Abbreviations: SFA, superficial femoral artery; PA, popliteal artery; TPT, tibioperoneal trunk.

**Table 5:** Procedural outcomes according to diabetic status.

Outcome	Non-Diabetic (*n* = 51)	Diabetic (*n* = 85)	*p*-Value
Technical success	37 (72.5%)	64 (75.3%)	0.71
Conversion to bypass	6 (11.8%)	10 (11.8%)	>0.05
Major amputation	2 (3.9%)	8 (9.4%)	0.32
In-hospital death	1 (2.0%)	3 (3.5%)	>0.05

## Data Availability

All relevant data are contained within the manuscript. The raw data supporting the conclusions of this article will be made available by the authors on request.

## Abbreviations

The following abbreviations are used in this manuscript:

ATA	Anterior tibial artery
CI	Confidence interval
CLTI	Chronic limb-threatening ischemia
DAPT	Dual antiplatelet therapy
DM	Diabetes mellitus
DSA	Digital subtraction angiography
EVT	Endovascular treatment
GLASS	Global Limb Anatomic Staging System
NOAC	Non-vitamin K oral anticoagulant
OR	Odds ratio
PAD	Peripheral artery disease
PA	Popliteal artery
PTA	Posterior tibial artery
PTA (procedure)	Percutaneous transluminal angioplasty
SFA	Superficial femoral artery
TPT	Tibioperoneal trunk
WIfI	Wound, Ischemia, and foot Infection classification
